# Effectiveness and safety of indirect moxibustion for the treatment of allergic rhinitis: A protocol for systematic review and meta-analysis of RCTs

**DOI:** 10.1097/MD.0000000000020911

**Published:** 2020-07-10

**Authors:** Ting Yuan, Yong Fu, Jun Xiong, Haifeng Zhang, Jun Yang, Xue Wang, Hao Fan, Yunfeng Jiang, Xiaohong Zhou, Kai Liao, Lingling Xu

**Affiliations:** a School of Acupuncture, Moxibustion and Tuina of Jiangxi University of Traditional Chinese Medicine; b Department of Acupuncture and Moxibustion, the Affiliated Hospital with Jiangxi University of Traditional Chinese Medicine, Nanchang, China.

**Keywords:** allergic rhinitis, indirect moxibustion, protocol, systematic review and meta-analysis

## Abstract

**Background:**

Allergic rhinitis (AR) is a common allergic disorder worldwide. Western medicine is not optimistic about the therapeutic effect of this disease. However, moxibustion can enhance vital energy or immunity through a great number of clinical trials. Thus, the aim of this systematic review and meta-analysis is to systematically evaluate the effectiveness and safety of indirect moxibustion for treating AR.

**Methods:**

We will conduct a comprehensive literature search in Medline, PubMed, Web of Science, Embase, the Cochrane Library, China National Knowledge Infrastructure Database, WanFang Database, Chinese Scientific Journal Database, and Chinese Biomedical Literature Database from inception to August 2020 without any language restriction. In addition, we will retrieve the unpublished studies and the references of initially included literature manually. Reviewers will identify studies, extract data, and assess the quality independently. The outcomes of interest include: total effective rate, total nasal symptom score, total non-nasal symptom score, rhinitis quality of life questionnaire, visual analog scale, laboratory indicators (i.e., serum levels of IgE, IgA, or IgG), and adverse events. Randomized clinical trials will be collected, methodological quality will be evaluated using the Cochrane risk-of-bias assessment tool, and the level of evidence will be rated using the Grading of Recommendations, Assessment, Development and Evaluation approach. Meta-analysis will be performed using RevMan 5.3.0 software. The heterogeneity test will be conducted between the studies, and *P* < .1 and I^2^ > 50% are the thresholds for the tests. We will utilize the fixed effects model or the random effects model according to the size of heterogeneity.

**Results:**

Because the review is ongoing, no results can be reported.

**Conclusions:**

The results of this review will provide reliable evidence for effectiveness and safety of indirect moxibustion for treating AR.

**Ethics and dissemination:**

Ethical approval is not required for this study. This systematic review and meta-analysis will be disseminated online and on paper to help guide clinicians.

**PROSPERO Registration number:**

CRD42019140944.

## 1 Introduction

Allergic rhinitis (AR) is a prevalent non-infectious inflammatory disease, which is caused by allergic individuals exposed to allergens and mainly characterized by allergic inflammation of nasal mucosa.^[1]^ In AR, the degranulation of IgE-mediated mast cell and the release of mediators cause a rapid response, resulting in sneezing, itchy palate, nasal blockage, runny nose, and nasal hemorrhoids, which may be related to eye symptoms, including itchy eyes, red eyes, watering, and burning sensation. An inflammatory reaction with eosinophilic infiltration may occur in later stages.^[2–4]^ The occurrence of AR has seriously influenced people's quality of life.^[5]^ AR is often accompanied by asthma attacks. It is clinically diagnosed based on detailed medical history, specialized examination, and laboratory tests to assess serum levels of specific immunoglobulins (IgE, IgA, or IgG).

The widespread of AR has remarkably attracted scholars’ attention. In recent years, the incidence of AR has been on a sharp rise, currently affecting about 10% to 20% of the world's population.^[6]^ Epidemiological surveys showed that the prevalence of AR in American adults ranges from 10% to 30%.^[7,8]^ Based on the AR and its impact on asthma (ARIA), the prevalence of AR is 40% to 50% in the general population of European countries and the USA^[7]^ and 4% to 38% in that of mainland China.^[9]^ Furthermore, AR is more likely to occur in individuals with atopy and family history of rhinitis, as well as in first-born children and immigrants.^[10–12]^ Although the disease is frequent among children, it accounts for about one-third of the adult rhinitis cases.

Nowadays, the treatment methods for AR mainly rely on Western medicine.^[13]^ The most commonly used drugs include corticosteroids, antihistamines, mast cell stabilizers, etc. Although these methods can temporarily alleviate nasal symptoms, they cannot completely cure AR. Moreover, the mentioned methods have shown remarkable side effects, such as drowsiness, dry mouth, and cardiac toxicity caused by antihistamines, etc.^[14]^ Acupuncture, as a non-pharmacological method, was recommended for AR patients by the latest guidelines for clinical practice published in 2015 in the USA.^[15,16]^ Non-pharmacological treatment, containing complementary and alternative medicine, for example, moxibustion, has shown significant clinical effects as well.^[17]^

Moxibustion is based on the theory of traditional Chinese medicine (TCM), and it typically bakes acupoints with burning moxa wool. It is mainly extracted from mugmoxa leaves, stimulating specific acupoints or parts of the body surface through the heat generated by hanging moxibustion, as well as stimulating the meridians and vital energy to regulate the viscera, so as to treat diseases.^[18]^ A previous research revealed that moxibustion has anti-allergic effects and can reduce the expression levels of signal transducer and activator of transcription 6 (STAT6), nuclear factor-κB (NF-κB), and inducible nitric oxide synthase (iNOS) in AR mice model.^[19]^ A number of clinical trials showed a remarkable efficiency of moxibustion in the treatment of AR.^[20,21]^ Indirect moxibustion is performed when an ignited moxa cone is placed on an insulated material and not directly in contact with skin. At present, air and paper are used as buffer layers for modern indirect moxa devices.^[22]^ This method not only has the moxibustion functions of warming meridian and dispersing cold, activating blood circulation, eliminating blood stasis and dispersing, preventing and caring for diseases, but also has the therapeutic effect of mild firepower, which is easy to accept by patients and has been extensively applied in clinical practice.^[23]^

Systematic reviews and meta-analyses could be advantageous for assessing clinical efficacy and developing clinical guidelines.^[24]^ However, to date, no systematic review or meta-analysis has concentrated on indirect moxibustion therapy for AR. The present systematic review was designed to assess the efficiency and safety of indirect moxibustion for treating AR patients. Intriguingly, several high-quality clinical trials have reported that Western medicine has serious adverse reactions, and its long-term application is prone to drug resistance, while indirect moxibustion has fewer adverse effects and higher safety. Hence, this systematic review and meta-analysis aimed to assess the quality of randomized controlled trials (RCTs), so as to evaluate the effectiveness and safety of indirect moxibustion for treating AR patients and better guide clinicians.

## 2 Methods

### 2.1 Study registration

This systematic review and meta-analysis will be performed according to the guidelines of the Cochrane Handbook for Systematic Reviews and Meta-Analysis Protocol.^[25]^ The protocol was previously registered in PROSPERO 2019 CRD42019140944, and it could be found at http://www.crd.york.ac.uk/PROSPERO/display_record.php?ID=CRD42019140944.

Any changes in the full text will be described.

### 2.2 Inclusion criteria

#### 2.2.1 Types of studies

We will include all eligible RCTs.

#### 2.2.2 Types of participants

Patients with all types of AR who were diagnosed with internationally recognized criteria (e.g., ARIA^[26]^) are eligible for inclusion. Patients’ age, gender, course of disease, syndrome type, and source of cases are not limited. Studies focused on AR combined with allergic asthma or allergic conjunctivitis and other allergic diseases will be excluded.

#### 2.2.3 Types of interventions

Studies that involved any form of indirect moxibustion (including moxibustion on ginger, moxibustion on garlic, moxibustion on salt, moxibustion on aconite cake, and compound moxibustion on medicine cake) as the sole treatment or as a major part of a combination therapy with other interventions (e.g., conventional drugs, etc) will be included. Importantly, no restrictions are placed on the number of acupoints, the method of moxibustion, duration, and frequency. However, studies will be excluded if indirect moxibustion is used as an adjuvant therapy.

#### 2.2.4 Types of comparator(s)/control

The comparators will include positive treatments (e.g., Western medicine, conventional therapy etc), no therapy, placebo or sham moxibustion. The specific forms of choice include the following items:

(1) Indirect moxibustion versus positive treatments;
(2) Indirect moxibustion + positive treatments versus positive treatments;
(3) Indirect moxibustion versus no therapy;
(4) Indirect moxibustion versus placebo;
(5) Indirect moxibustion versus sham moxibustion.

#### 2.2.5 Types of outcome measures

##### 2.2.5.1 Primary outcomes

The total effective rate and total nasal symptom score^[27]^ (including rhinorrhea, nasal itching, nasal obstruction, and sneezing) will be used as primary outcomes.

##### 2.2.5.2 Secondary outcomes

(1) Total non-nasal symptom score^[27]^;
(2) Rhinitis quality of life questionnaire^[28]^;
(3) Visual analog scale;
(4) Laboratory indicators: serum levels of IgE, IgA, or IgG;
(5) Adverse events.

### 2.3 Exclusion criteria

Duplicate detection and republished literature; indirect moxibustion + other non-pharmacological treatment; experts’ experience or case reports; theoretical research or experimental research; unclear diagnostic criteria and efficacy evaluation criteria; conference papers and incomplete data of the results.

### 2.4 Search methods

We will conduct a comprehensive literature search in Medline, PubMed, Web of Science, Embase, the Cochrane Library, China National Knowledge Infrastructure Database, WanFang Database, Chinese Scientific Journal Database, and Chinese Biomedical Literature Database from inception to August 2020 without any language restriction. In addition, we will also retrieve the unpublished studies and the references of initially included literature manually. Searches will be re-run prior to the final analysis. The research will start following the PRISMA protocol. The main keywords include: “indirect moxibustion”, “allergic rhinitis”, and “RCT”. The search will be restricted to human subjects, while there is no restriction on any specific languages. The proposed search strategy for PubMed is presented in Table 1.

**Table 1:**
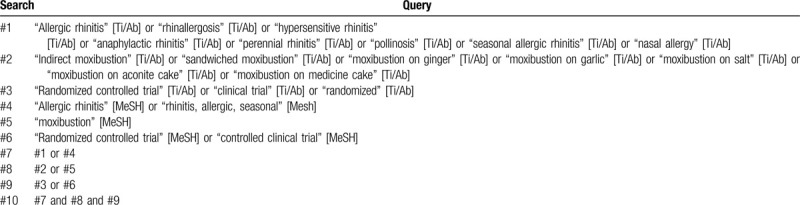
PubMed: will be searched in: August 2020.

### 2.5 Selection process

We will include qualified trials according to Cochrane Collaborative System Evaluator's Handbook 5.2.0.^[29]^ The specific steps are as follows:

(1) The literature will be obtained through comprehensive retrieval of the specific databases and imported into the document management software NoteExpress 3.0, and the repeated documents will be excluded from each database.
(2) We will read the titles and abstracts preliminarily excluding the literature irrelevant to this study.
(3) The selected clinical studies will be downloaded, and the full text will be screened.
(4) The number of included RCTs will be determined by screening the literature of included studies according to inclusion and exclusion criteria.

**Figure 1:**
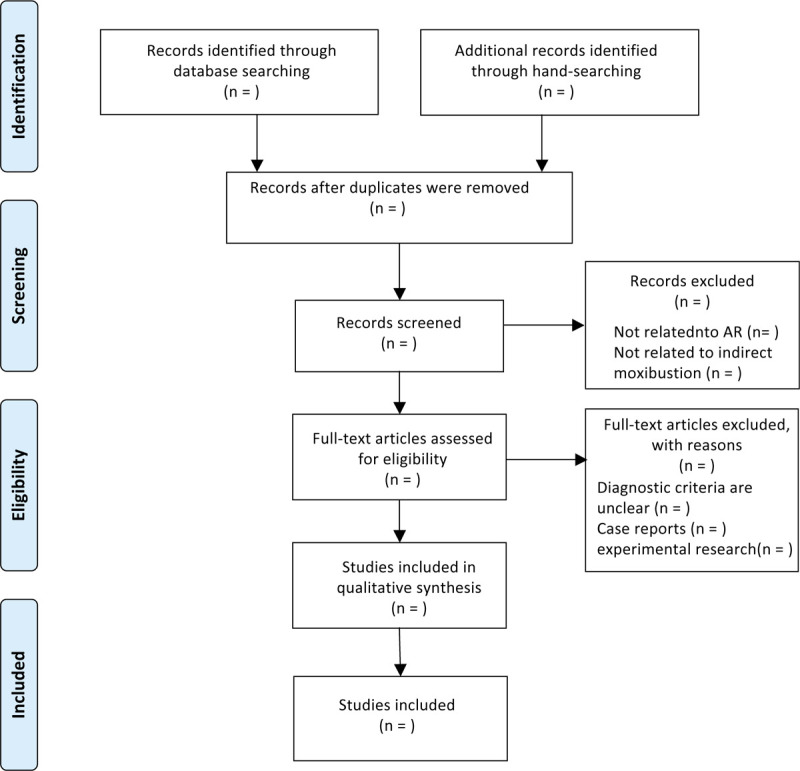
Flowchart of literature selection.

Three independent researchers (TY, HF, and WX) will follow the procedure strictly. If there is any disagreement, it will be resolved by the fourth researcher (JY). The flowchart of selection process is illustrated in Figure 1.

### 2.6 Data extraction and management

We will establish a normal data extraction table in line with PICOST. Before the formal data extraction, pre-extraction will be conducted twice to ensure the smooth progress of the formal extraction. Data extraction is carried out independently by 3 researchers (TY, HF, WX) and cross-checked repeatedly. In case of disagreement, a tripartite discussion will be conducted to reach an agreement or a fourth investigator (JY) will assist in the determination. Meanwhile, intention-to-treat (ITT) analysis will be applied to the missing data. We will use Excel 2007 for data extraction. Relevant contents of data extraction include: title, author, publication time, average age, sample size, disease type, course of treatment, intervention measures, control measures, adverse reactions, outcome indicators, etc. When important data in the literature are missing or incomplete, the corresponding author will be contacted by phone or email.

### 2.7 Assessment of the methodological quality

The methodological quality of qualified trials will be evaluated by using the Cochrane risk assessment tool^[30]^ according to Cochrane Reviewer's Handbook 5.0. It includes 7 aspects: random sequence generation, allocation concealment, blinding of participants or doctors, blinding of outcome evaluator, incomplete outcome data, selective outcome reporting, and other bias. High (H), low (L), and unclear (U) will be selected to assess the degree of risk of bias in each item. The quality of the evaluation results included in the test will be cross-examined by 3 evaluators (TY, HF, and XW). If necessary, the corresponding author will be contacted to clarify the issues. Any disagreements will be resolved through discussion or consultation with a fourth reviewer (JY).

### 2.8 Level of evidence

The quality of evidence of the included RCTs will be assessed by Grading of Recommendations, Assessment, Development and Evaluation approach using GRADEprofiler 3.6 software. RCTs start with high level of evidence. The level of evidence will be assessed as follows: high, moderate, low, and very low. If there are limitations on risk of bias, inconsistency, imprecision, indirectness, and publication bias, we will decrease 1 or 2 levels.^[31]^ Two reviewers (TY and HF) will evaluate the level of evidence independently. In case of disagreement, a discussion will be conducted to reach an agreement or a fourth reviewer (JY) will assist in the determination.

### 2.9 Data synthesis

#### 2.9.1 Measures of treatment effect

Meta-analysis will be performed using RevMan 5.3.0 software. The data are summarized using relative risk (RR) with 95% confidence intervals (CI) for binary outcomes or mean difference with 95% CI for continuous outcomes. If different measurement scales are used, standardized mean difference analyses will be carried out. If the missing data cannot be achieved by contacting the corresponding author, the existing data will be analyzed.

#### 2.9.2 Heterogeneity

Forest plots will be constructed, and the heterogeneity between the qualified studies is tested. Chi-squared test and I^2^ value are used to assess the degree of heterogeneity. When *P* < .1, I^2^ > 50%, no heterogeneity is considered among the trials, and the fixed effects model will be used for statistical analysis; otherwise, the random effects model will be used. When the clinical heterogeneity between the 2 studies is large, only descriptive analysis will be undertaken.

#### 2.9.3 Publication bias

When the number of included studies is > 10, the Egger test is used for potential publication bias.^[32]^ If *P* > .05, the possibility of publication bias is small.

#### 2.9.4 Subgroup analysis

If the necessary data are available, we will conduct subgroup analyses to assess heterogeneity according to the different control measures (e.g., Western medicine, no treatment or placebo moxibustion, etc), period of treatment, and outcome measures.

#### 2.9.5 Sensitivity analysis

The goal of sensitivity analysis is to identify the sources of heterogeneity and confounding factors. If the trials’ data are sufficient, sensitivity analysis will be conducted by using the STATA 14.0 software.

## 3 Discussion

In the discussion part of the full text, we will summarize and analyze the research results of indirect moxibustion therapy for AR from the following aspects:

(1) Indirect moxibustion intervention mechanism;
(2) Main findings of indirect moxibustion effects;
(3) Quality of evidence;
(4) Discussion of heterogeneity;
(5) Limitations and strength of the study; and
(6) Conclusion.

At present, AR is mainly treated with antihistamines and intranasal topical glucocorticoids in the nose.^[33]^ Topical intranasal glucocorticoids are the most commonly used therapy for moderate and severe AR in Western medicine.^[34]^ However, prolonged use of glucocorticoids can cause serious side effects, such as nasal dryness and nosebleed, and the incidence can be as high as 20%.^[35]^ Moreover, Western medicine is not effective for certain patients with moderate to severe AR.^[36]^ Although desensitization treatment could effectively improve the symptoms of AR, it has a number of disadvantages, including long course of disease, easy recurrence, and remarkable side effects. Hence, scholars should further concentrate on non-drug therapy to reduce the clinical symptoms and adverse reactions of AR.

Indirect moxibustion at 2 acupoints (CV 4 and CV 8) has an antioxidative capacity. It can significantly decrease the serum levels of total ROS and MDA, while increase the TAC level and catalase activity after moxibustion.^[37]^ Nowadays, in view of the rare reports on the exact mechanism of indirect moxibustion for treating AR, it is suggested to intensify the clinical research on indirect moxibustion for AR in order to provide evidence-based medicine programs for clinicians better.

This study has a number of limitations: firstly, due to the limitations of retrieval conditions, there is no way to ensure that all relevant RCTs can be included; secondly, there is a certain language bias in terms of searching only Chinese and English databases; thirdly, if we fail to contact the corresponding author, we may not be able to obtain the missing data; finally, in the process of indirect moxibustion treatment, the blind method is difficult to implement, which may also cause bias.

## 4 Conclusion

This is the first protocol for a systematic review designed to evaluate the effectiveness and safety of indirect moxibustion for AR. And this study will include comprehensive assessment of methodological quality and the level of evidence. Therefore, we speculate that our findings will provide reliable evidence-based medical evidence for indirect moxibustion treatment of AR. In addition, we expect that more research to be conducted in the future to elucidate the shortcomings of the present meta-analysis.

## Author contributions

**Conceptualization:** Ting Yuan, Yong Fu, Jun Xiong.

**Data curation:** Ting Yuan, Hao Fan, Xue Wang, Jun Yang.

**Formal analysis:** Ting Yuan, Xue Wang, Jun Yang.

**Investigation:** Jun Xiong, HaiFeng Zhang.

**Methodology:** Jun Xiong, Yong Fu, HaiFeng Zhang.

**Software:** YunFeng Jiang, XiaoHong Zhou, LingLing Xu, Kai Liao.

**Supervision:** Jun Xiong, Hao Fan.

**Writing – original draft:** Ting Yuan, Yong Fu, Jun Xiong.

**Writing – review & editing:** HaiFeng Zhang, Jun Yang, YunFeng Jiang, XiaoHong Zhou, Kai Liao, LingLing Xu.
